# Spatial interactions between sympatric carnivores: asymmetric avoidance of an intraguild predator

**DOI:** 10.1002/ece3.1561

**Published:** 2015-06-19

**Authors:** Shaun M Grassel, Janet L Rachlow, Christopher J Williams

**Affiliations:** 1 Department of Fish and Wildlife Sciences, University of IdahoMoscow, Idaho, 83844-1136; 2 Department of Wildlife, Fish and Recreation, Lower Brule Sioux TribeP.O. Box 246, Lower Brule, South Dakota, 57548-0246; 3 Department of Statistical Science, University of IdahoMoscow, Idaho, 83843-1104

**Keywords:** Coexistence, competition, foraging trade-offs, interspecific interactions, intraguild predation, intrasexual territoriality

## Abstract

Interactions between intraguild species that act as both competitors and predator–prey can be especially complex. We studied patterns of space use by the black-footed ferret (*Mustela nigripes*), a prairie dog (*Cynomys* spp.) specialist, and the American badger (*Taxidea taxus*), a larger generalist carnivore that competes for prairie dogs and is known to kill ferrets. We expected that ferrets would spatially avoid badgers because of the risk of predation, that these patterns of avoidance might differ between sexes and age classes, and that the availability of food and space might influence these relationships. We used location data from 60 ferrets and 15 badgers to model the influence of extrinsic factors (prairie dog density and colony size) and intrinsic factors (sex, age) on patterns of space use by ferrets in relation to space use by different sex and age categories of badgers. We documented asymmetric patterns of avoidance of badgers by ferrets based on the sex of both species. Female ferrets avoided adult female badgers, but not male badgers, and male ferrets exhibited less avoidance than female ferrets. Additionally, avoidance decreased with increasing densities of prairie dogs. We suggest that intersexual differences in space use by badgers create varying distributions of predation risk that are perceived by the smaller carnivore (ferrets) and that females respond more sensitively than males to that risk. This work advances understanding about how competing species coexist and suggests that including information on both intrinsic and extrinsic factors might improve our understanding of behavioral interactions between sympatric species.

## Introduction

Interactions between sympatric taxa help shape the structure of ecological communities because they can influence the demography, distribution, and behavior of species within communities (Holt and Polis 1997; Berger and Gese 2007). When sympatric species use similar resources, interspecific interactions are likely to include 1 of 2 forms of competition (Linnell and Strand 2000). Exploitative competition occurs when 1 species is more efficient than its competitors at exploiting available resources (Case and Gilpin 1974). Alternatively, interference competition occurs when less efficient competitors directly interfere with their more specialized competitors, which can result in the direct displacement of competitors (Case and Gilpin 1974), kleptoparasitism (Creel 2001; Creel et al. 2001), or intraguild predation (Polis et al. 1989; Palomares and Caro 1999). The latter can be a significant source of mortality for some species, and such interactions are of particular concern when threatened or endangered species are killed by competitors (Palomares and Caro 1999; Linnell and Strand 2000).

The potential for competition between sympatric carnivores using similar resources is largely determined by the extent of spatial overlap (Kitchen et al. 1999; Palomares and Caro 1999; Sergio et al. 2003). Therefore, to minimize interference competition and the risk of intraguild predation, the strategy of a subordinate competitor often includes avoidance of the dominant species (Case and Gilpin 1974; Robinson and Terborgh 1995; Atwood and Gese 2010). Among mammalian carnivores, for example, red foxes (*Vulpes vulpes*) avoid coyotes (*Canis latrans*) by establishing home ranges outside of the areas used by coyotes (Major and Sherburne 1987; Theberge and Wedeles 1989). Bobcats (*Lynx rufus*) also avoid interactions with coyotes at fine spatial scales by establishing core use areas that do not overlap with coyote core areas (Thornton et al. 2004). Similarly, home ranges of coyotes often are located along the edges of wolf (*Canis lupus*) territories or in the matrix between territories of neighboring wolf packs (Arjo and Pletcher 1999). Availability of spatial refugia can be important for coexistence of competitors, including intraguild prey and predators (Sergio et al. 2003).

Within Mustelidae, there are several sympatric species with similar niches that have developed mechanisms to coexist. Mink (*Mustela vison*) and river otters (*Lutra canadensis*) persist in coastal marine environments by partitioning diets and also habitat by the amount of wave action and over-story cover (Ben-David et al. 1995). Similar results were reported for mink and Eurasian otters (*Lutra lutra*) coexisting in riverine environments (Bonesi and Macdonald 2004; Bonesi et al. 2004). In North America, short-tailed weasels (*M. erminea*) reduce the use of their preferred habitat when occupied by long-tailed weasels (*M. frenata*), a dominant guild member that has been documented to kill and eat short-tailed weasels (Gamble 1980; St-Pierre et al. 2006). In Europe, the least weasel (*M. nivalis*) and short-tailed weasel coexist because the subordinate least weasel can avoid confrontations with the dominant generalist using the tunnels and runways of voles (*Microtus* spp.), its primary prey (King and Moors 1979; Aunapuu and Oksanen 2003).

**Figure 1:**
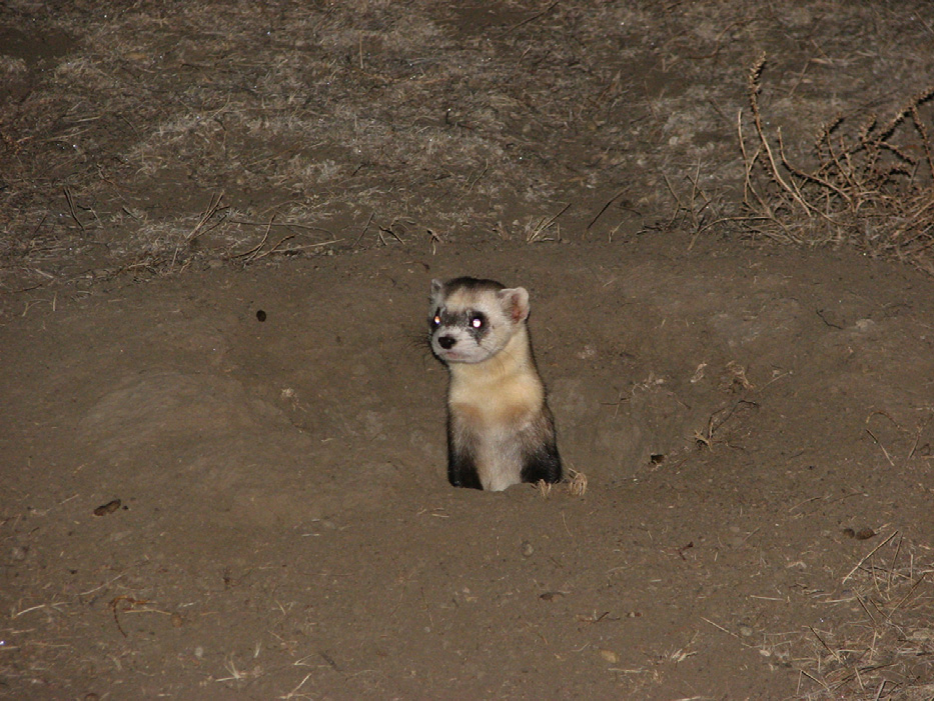
Black-footed ferret (*Mustela nigripes*).

Similarly, black-footed ferrets (*M. nigripes*; hereafter ferrets; Fig.1) and American badgers (*Taxidea taxus*; hereafter badgers) coexist in prairie dog (*Cynomys* spp.) ecosystems in North America. Ferrets are obligate specialists that are dependent on prairie dogs for food and reside within burrows created by prairie dogs (Biggins 2006). In contrast, badgers are often considered generalist foragers that are common in a variety of grassland-dominated habitats where they consume a diversity of vertebrates (primarily mammals), invertebrates, and plant material (Messick and Hornocker 1981; Lampe 1982; Goodrich and Buskirk 1998; Sovada et al. 1999). When available, prairie dogs often are the most common food item in the diet of badgers (Goodrich and Buskirk 1998), and both ferrets and badgers select areas with high densities of active burrows where prairie dogs are relatively abundant (Biggins et al. 1993; Eads et al. 2011, 2013; Jachowski et al. 2011). Overlap in diet and fine-scale patterns of resource selection likely results in strong competition between the two carnivores. Badgers are known to kill ferrets (Biggins et al. 1999, 2006a, 2011b) and excavate prairie dog burrows more often in areas recently used by a ferret, suggesting that badgers actively hunt ferrets, steal prey from ferrets, or both (Eads et al. 2013).

We evaluated the influence of intrinsic factors (sex and age) and extrinsic factors (prairie dog density and prairie dog colony size) on space use by ferrets in relation to space use by badgers. We hypothesized that (1) ferrets would reduce the potential for intraguild predation by spatially avoiding badgers, and we expected that; (2) adult female badgers would have the greatest influence on space use by ferrets because female badgers are more attuned to the distribution of prey resources and have smaller territories than males (Messick and Hornocker 1981; Minta 1993; Goodrich and Buskirk 1998), and thus, female badgers likely create a more predictable presence that intraguild prey might attempt to avoid; (3) female ferrets would avoid badgers more than males because of the need to minimize the risk of predation on neonates; (4) adult ferrets would exhibit greater avoidance of badgers than juvenile ferrets because social dominance allows adults to select areas with lower risk; (5) the availability of food (prairie dogs) would influence space use by ferrets, and we expected that ferrets would trade off increased risk of predation by badgers for access to food by exhibiting less spatial avoidance of badgers when densities of prairie dogs were relatively low; and (6) the availability of spatial refugia would influence space use by ferrets, and we predicted that ferrets occupying small colonies would exhibit less spatial avoidance of badgers relative to ferrets on large colonies because the lack of space would prevent ferrets from maintaining safe distances from areas of high use by badgers.

However, because badgers select the same habitat patches as ferrets and might be attracted to ferrets (Eads et al. 2013), our alternate hypothesis was that at fine spatial scales, space use patterns of ferrets and badgers would overlap. Coexistence of ferrets and badgers at fine spatial scales would likely be facilitated, in part, by the relatively high efficiency of ferrets to prey on prairie dogs as compared to badgers (i.e., exploitative competition) and by the selection of burrow systems with multiple openings, which likely lowers the risk of predation by badgers (Biggins 2012). Understanding factors that shape spatial interactions between generalists and specialists that have overlapping niches can provide insight into the function and structure of biotic communities.

## Materials and Methods

### Study area

We conducted our study from 2008 to 2010 on two native prairie grasslands, the Lower Brule Indian Reservation and Buffalo Gap National Grasslands, located approximately 250 km apart in central and southwestern South Dakota, USA, respectively. At the Lower Brule Indian Reservation, our research was conducted on the Fort Hale Bottom prairie dog complex (469857E, 4868645N NAD 83 Zone 14N; hereafter Lower Brule) located in a fragmented mixed-grass prairie dominated by western wheatgrass (*Pascopyrum smithii*), green needlegrass *(Nassella viridula*), buffalo grass (*Bouteloua dactyloides*), and needle-and-thread (*Hesperostipa comata*). The area is primarily used for livestock grazing, but cultivated agriculture also is common. In 2007, the Lower Brule study site contained 25 prairie dog colonies totaling 642.5 ha (range = 0.1–209.1 ha, 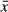 = 23.8). Our research focused on six prairie dog colonies that ranged in size from 9.3 to 207.7 ha (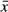 = 53.8 ha). Previous work using visual count methods (Severson and Plumb 1998) estimated densities of prairie dogs at 44.7/ha and 56.1/ha in 2004 and 2005, respectively (Lower Brule Sioux Tribe, unpubl. data). This population of ferrets was established when captive-born and wild-born ferrets were translocated from captive breeding facilities and other recovery sites in 2006 and 2007. During our study, the ferret population was comprised primarily of wild-born offspring and a few wild-born ferrets from the original releases.

Our research at the Buffalo Gap National Grasslands occurred on the Heck Table prairie dog complex (699205E, 4843996N NAD 83 Zone 13N; hereafter Buffalo Gap). The site is a mixed-grass prairie dominated by western wheatgrass, buffalograss, blue grama (*Bouteloua gracilis*), and prickly pear cactus (*Opuntia polyacantha*), and it is nearly devoid of tree and shrub species (Schroeder 2007; Livieri and Anderson 2012). Primary land uses for the area are cattle grazing and recreation (Livieri and Anderson 2012). In 2007, the Buffalo Gap study site supported 31 colonies of prairie dogs that totaled 1192.9 ha (range = 0.2–284.6 ha, 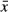 = 38.5 ha). Our research focused on 2 colonies that were 250.3 and 284.6 ha in size. Densities of prairie dogs were estimated at 27.9/ha in this area in 1999 (Biggins et al. 2011a) and 41.0/ha on nearby colonies in the Conata Basin in 2007 (Biggins et al. 1993; Eads et al. 2011). This population of ferrets was established in 1999 with the release of ferrets reared at captive breeding facilities and wild-born ferrets translocated from the Conata Basin. During our study, all ferrets were wild-born.

### Ferret and badger monitoring

We located ferrets for capture and marking and for subsequent relocations by conducting nighttime spotlight surveys (Biggins et al. 2006b), which consisted of an observer in a vehicle using a roof-mounted spotlight to systematically search entire prairie dog colonies for the reflective eye-shine of ferrets. At Lower Brule, we captured ferrets using wire cage traps (91.5 × 10 × 10 cm) placed into burrow entrances, anesthetized individuals in a mobile laboratory setting using isoflurane, and implanted each with Passive Integrated Transponders (PIT; AVID® Microchip I.D. Systems, Folsom, LA). A contractor for the U. S. Forest Service marked ferrets at Buffalo Gap. Ferrets detected visually during subsequent surveys were identified using automated PIT tag readers placed at the opening of the burrow (Fagerstone and Johns 1987; Stoneberg 1996; Biggins et al. 2006b), and their locations were recorded using handheld GPS receivers. Individuals that anesthetized ferrets received specialized training and authorization from the U. S. Fish and Wildlife Service (permit #TE-131398).

We searched prairie dog colonies to locate badgers for capture. We set padded leg-hold traps (#3 coil spring, Victor Soft Catch, Woodstream Corp., Lititz, PA) at the entrance of occupied badger burrows. Trapped individuals were chemically immobilized with an intramuscular injection of ketamine hydrochloride and xylazine hydrochloride (15 mg/kg body weight and 1.5 mg/kg, respectively; Goodrich and Buskirk 1998), marked with a PIT, and fitted with a harness-style radio transmitter weighing approximately 100–110 g (Advanced Telemetry Systems, Isanti, MN). The anesthetic effects of xylazine hydrochloride were reversed by intravenous injection of yohimbine (0.125 mg/kg; Goodrich and Buskirk 1998). Relocations of telemetered badgers were primarily obtained at night.

We monitored ferrets and badgers year-round at Lower Brule and seasonally at Buffalo Gap. Monitoring of both species occurred during November 2008–November 2010 at Lower Brule and May–November 2009 at Buffalo Gap. We considered juvenile badgers and ferrets independent of their parents on 1 September of the year of their birth and did not include locations of juveniles prior to this date in our analyses. Juveniles were considered adults on 1 April of the year after their birth.

### Prairie dog colony size and density

We mapped prairie dog colonies at Lower Brule in August 2007, and personnel of the U.S. Forest Service mapped colonies at Buffalo Gap during May–September 2007 using Trimble® GeoXM™ GPS receivers (1-m accuracy) while driving an ATV or walking along the perimeters of the colonies. Edges of colonies were distinguished by the distinct difference in vegetation height caused by foraging and vegetation clipping behavior of black-tailed prairie dogs (Koford 1958; Hoogland 1995). We imported data into ArcGIS (v. 9.3; ESRI, Redlands, CA) to estimate colony size and to conduct spatial analyses.

We captured and marked prairie dogs to estimate relative densities at the colony level. We conducted trapping within randomly selected 0.5-ha plots during June–July 2008 at the Lower Brule site, June 2009 at both study sites, and also during June 2010 at the Lower Brule site. At Lower Brule, we established four trapping plots on each of six prairie dog colonies, which resulted in sampling 1–22% of the area of each colony. At Buffalo Gap, we established 12 plots on the smaller colony (250.3 ha) and 16 on the larger colony (284.6 ha), which resulted in sampling 2–3% of each colony. Each plot contained 61 wire box traps (Tomahawk Livetrap Company, Tomahawk, WI; Tru-Catch Traps, Belle Fourche, SD) baited with grain and molasses pellets spaced 10 m apart in a 7 × 7 design with 12 additional traps placed in areas with high densities of burrow openings to minimize trap saturation. We prebaited plots for 3 days prior to trapping, and we set and checked traps twice each day for four consecutive days and checked them once on the 5th day (i.e., nine trapping sessions). We marked prairie dogs on initial capture with hair dye in 2008, by clipping a toe-nail in 2009, and by clipping a toe-nail and applying individually numbered ear tags in 2010. An index of relative density for each colony was obtained each year by averaging the total number of individuals marked per plot across all plots. We attempted to improve these estimates using accumulation curves and modifications to Peterson–Lincoln estimation methods (Schnabel 1938; Chapman 1951), but these methods produced unrealistic estimates. Therefore, we used the average number marked per plot to provide a relative index of density of prairie dogs on the study colonies rather than an estimate of population density. All animal use methods were approved by the University of Idaho Animal Care and Use Committee (protocol #2008-26) and conformed to the guidelines for use of wild mammals in research established by the American Society of Mammalogists (Sikes et al. 2011).

### Spatial analyses

Our study design followed a second-order selection analysis of habitat use versus availability (Johnson 1980). We buffered all ferret locations with an 80-m-radius circular plot to represent areas used by ferrets, and we generated three random locations per ferret location, which were cast outside of the buffered use areas but within the prairie dog colony where that ferret was located. If a ferret used >1 colony, which did not occur often, the number of random locations included for each colony was proportional to the number of ferret relocations per colony.

We generated utilization distributions (UDs) for each badger to estimate the relative intensity of use across space (Van Winkle 1975; Kernohan et al. 2001). We used fixed-kernel estimation (Worton 1989) with a likelihood cross-validation smoothing parameter to estimate individual 99% UDs with a grid cell size of 30 × 30 m using the Hawth’s Tools extension (Beyer 2004) in ArcGIS and Animal Space Use (v. 1.3; Horne and Garton 2009). We estimated UDs for each badger for which we recorded ≥27 locations. For juvenile badger UDs, we only used locations collected between 1 September of their birth year and 31 March of the following year. For badgers that were radio-tracked as juveniles and adults, a juvenile and adult UD were created when there were an adequate number of locations for each age category.

We determined a value for intensity of badger use for both random and available ferret locations by extracting the raster value from overlapping badger UDs in ArcGIS. If a location was within the UD of >1 badger, we summed the values for intensity of badger use and developed cumulative estimates for adult female badgers, adult male badgers, and all badgers. Our analyses only included ferrets that had ≥3 locations within a badger UD.

### Statistical analyses

We evaluated relative support for models of intrinsic and extrinsic factors shaping space use by ferrets relative to intensity of space use by badgers. We modeled characteristics predicting used and unused sites with logistic regression with a repeated measure on individual ferrets to control for individual variability using the GENMOD procedure in SAS (v. 9.3; SAS Institute Inc., Cary, NC). We used sex and age of each ferret as intrinsic predictor variables and colony-level attributes (colony size and relative density of prairie dogs) as extrinsic predictor variables. We included the effect of study site in some models because features unique to each study site (e.g., other predators, alternative prey, etc.) that we did not measure also might influence spatial interactions between ferrets and badgers.

Our primary focus was the evaluation of spatial interactions, and consequently, we modeled the probability of use by ferrets in relation to the space use by badgers. Our candidate models included the intrinsic and extrinsic predictor variables and their 2-way interactions with the variable that represented intensity of badger use. Interactions between the intensity of badger use and colony-level attributes (colony size and relative density of prairie dogs) would be a consequence of how badgers use prairie dog colonies, which would vary spatially across colonies. To determine whether sex or age categories of badgers influenced ferrets differently, we developed a set of models for adult female badgers, adult male badgers, and all badgers. Each set contained 12 candidate models that included the effects of study site, sex and age of the ferret, relative density of prairie dogs, size of prairie dog colonies, and the variable for relative intensity of badger use. Each set of models only differed by the variable that represented the intensity of badger use (i.e., adult females, adult males, or all badgers). Because of small sample sizes, we did not evaluate models that contained an age*sex covariate. The effect of badger presence was modeled as interactions with the other variables, and consequently, must be interpreted in relation to the other variables. We compared models using quasilikelihood under the independence model criterion (QIC; Pan 2001; Burnham and Anderson 2002; Hardin and Hilbe 2003) to identify the most parsimonious models that fit the observed data.

## Results

We recorded 707 locations from 15 badgers (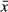 = 47.1, range = 27–84, SD = 21.0): six adult females, one juvenile female, four adult males, three juvenile males, and one female that was radio-tracked as a juvenile and as an adult. We recorded 639 locations from 60 ferrets (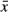 = 10.7, range 3–34, SD = 8.2): 16 adult females, 15 juvenile females, 11 adult males, seven juvenile males, and 11 ferrets (four females and seven males) that had locations recorded as both juveniles and as adults. Our analyses included prairie dog colonies that ranged in size from 9.3 to 284.6 ha (*n* = 8, 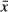 = 107.1 ha, SD = 119.9 ha), and indices of relative densities of prairie dogs that averaged 9.0 to 66.5 per 0.5-ha plot (*n* = 17 colony-years, 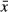 = 29.3, SE = 3.7).

As expected, adult female badgers more strongly influenced space use by ferrets than other sex and age categories. Only two of the candidate models evaluating space use by ferrets were included in the 95% confidence set (i.e., models with ≥95% of the weight, *W*_*i*_, based on the QIC values), and both included intensity of use by adult female badgers rather than adult males or all badgers (Table1). Models with other sex and age categories of badgers were >133 ΔQIC from the top model and received <0.0001 percent of the model weight, indicating markedly poorer fits to the data.

**Table 1:** Ninety-five percent confidence set of the candidate models of factors influencing probability of space use by black-footed ferrets relative to space use by badgers on colonies of black-tailed prairie dogs within the Lower Brule Indian Reservation and Buffalo Gap National Grasslands, South Dakota, during 2008–2010

Model	QIC^1^	ΔQIC^2^	*W*_*i*_ 3
AFB Sex PD Site AFB^*^Sex AFB^*^PD AFB^*^Site	1935.95	0	0.947
AFB Sex PD Colony AFB^*^Sex AFB^*^PD AFB^*^Colony	1942.14	6.19	0.043

1 QIC is the quasilikelihood under the independence model criterion.

2 ΔQIC is the difference from the model with the lowest QIC value.

3 *W*_*i*_ is the QIC weight of the model.

Variables for models: adult female badger intensity of use (AFB), ferret sex (Sex), relative prairie dog density (PD), colony size (Colony), and study site (Site). Two-way interactions are denoted by an asterisk.

**Figure 2:**
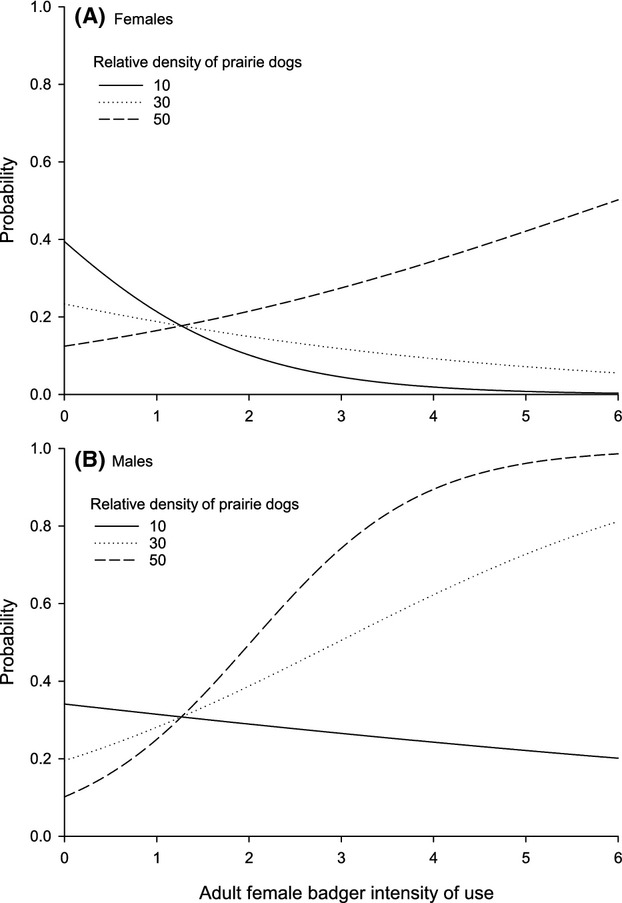
Probability of space use by female (A) and male (B) black-footed ferrets (*Mustela nigripes*) as a function of relative intensity of use by adult female badgers (*Taxidea taxus*) and relative density of black-tailed prairie dogs (*Cynomys ludovicianus*). The lines representing prairie dog densities ranging from 10 to 50 individuals per 0.5-ha plots were within the range of variation documented across our study sites. Adult female badger intensity of use is an index that was within the range of variation documented across our study sites. Sex of ferrets and relative density of prairie dogs were both significant factors (Table2).

Both intrinsic and extrinsic factors shaped the spatial interactions of ferrets and badgers. Patterns of avoidance of adult female badgers differed between the sexes of ferrets and were influenced by the relative density of prairie dogs. As we predicted, female ferrets exhibited stronger spatial avoidance of adult female badgers than males. Both candidate models in the 95% confidence set included sex of ferrets and relative density of prairie dogs, each interacting with the intensity of use by adult female badgers (Table1). Parameter estimates from the top model with >94% of the model weight indicated that both of these factors were significant and that avoidance of badgers by female ferrets was nearly twice that of males (Table2). Ferrets spatially avoided badgers on colonies with lower densities of prairie dogs, but contrary to our expectations, avoidance decreased with increasing densities of prairie dogs (Fig.2).

**Table 2:** Parameter estimates for the top model evaluating space use by black-footed ferrets relative to space use by badgers on the Lower Brule Indian Reservation and Buffalo Gap National Grasslands, South Dakota, during 2008–2010

Parameter	Coefficient estimate	Standard error	95% Confidence limits		*P* value
Intercept	−0.279	0.379	−1.022	0.463	0.461
AFB	−0.420	0.597	−1.589	0.748	0.482
Sex (F)	0.232	0.141	−0.045	0.508	0.101
PD	−0.038	0.014	−0.066	−0.010	0.008
Site (BG)	−0.549	0.294	−1.126	0.027	0.062
AFB^*^Sex (F)	−0.754	0.282	−1.308	−0.201	0.008
AFB^*^PD	0.030	0.013	0.004	0.055	0.024
AFB^*^Site (BG)	1.267	0.986	−0.666	3.200	0.199

Variables for models: adult female badger intensity of use (AFB), ferret sex (Sex), relative prairie dog density (PD), colony size (Colony), and study site (Site). Two-way interactions are denoted by an asterisk.

The top model also included the variable for the interaction between study site and intensity of use of adult female badgers, suggesting that patterns of avoidance of adult female badgers by ferrets differed between study sites (Table1). However, the confidence intervals for the study site parameter estimate encompassed 0 in that model, suggesting that differences between study sites had a relatively weak influence on avoidance of badgers by ferrets in comparison with ferret sex and relative density of prairie dogs.

Neither the size of prairie dog colonies nor the age class of ferrets appeared to have a marked effect on spatial interactions between ferrets and badgers in our study. Although colony size was in 1 model in the 95% confidence set (Table1), the confidence interval for that parameter estimate encompassed 0, and the model was not considered a competing model (ΔQIC = 6.19).

## Discussion

Our results revealed complex, asymmetric patterns of spatial overlap between 2 sympatric carnivores that interact as potential competitors and as potential prey and predators. As expected, badgers did not influence space use by ferrets equally across sex and age categories; adult female badgers had a greater influence on the movements of ferrets than adult male badgers or all badgers combined. Ferret response to badgers likewise differed between sexes, with females responding more strongly than males. These differences suggest that the spatial interactions of sympatric species might be difficult to predict without information about how intrinsic factors shape behavioral interactions among individuals.

**Figure 3:**
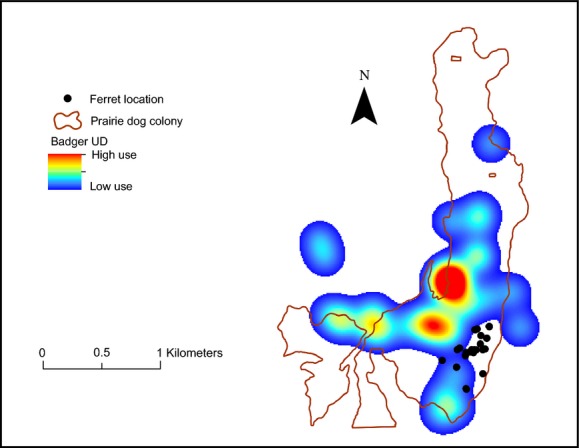
Locations of an adult black-footed ferret in relation to the UD of an adult female badger on the Buffalo Gap National Grasslands.

One explanation for the difference in apparent influence of female and male badgers on space use by ferrets is that intersexual differences in space use result in spatially concentrated versus dispersed predation risk. Badgers exhibit distinct intersexual differences in social behavior and space use: Female spatial organization is shaped largely by the distribution of food resources while male spatial patterns are determined primarily by the distribution of females, which results in the territories of females being smaller than those of males (Messick and Hornocker 1981; Minta 1993; Goodrich and Buskirk 1998). Adult female badgers preferentially use prairie dog colonies when available, establish their territories within colony boundaries, and prey primarily on prairie dogs (Goodrich and Buskirk 1998). Within prairie dog colonies, adult female badgers might be considered an “organizing force” and the core area of their territory may create an “influence field” that intraguild prey might attempt to avoid (Forman 1995). This relationship has been in observed in other predator–prey systems. For example, eagle owls (*Bubo bubo*) are considered to have an organizing force on black kites (*Milvus migrans*), which minimize their predation risk by establishing territories outside of the influence of owl pairs (Sergio et al. 2003). Similarly, the presence of tiger sharks (*Galeocerdo cuvier*), even at low densities, influenced the spatial patterns of bottlenose dolphins (*Tursiops aduncus*), such that dolphins avoid using areas that were used by tiger sharks even though food was abundant (Heithus and Dill 2002). Wolf packs can influence spatial patterns of both potential prey and intraguild competitors. White-tailed deer (*Odocoileus virginianus*; Mech 1977), elk (*Cervus elaphus;* Fortin et al. 2005), and coyotes (Arjo and Pletcher 1999) minimize risk of predation by avoiding areas used by wolves. In our study, the more restricted pattern of space use that characterizes adult female badgers likely creates a predator presence that is more predictable in space and time relative to males, and ferrets can likely detect and respond more readily to this relatively static cue of predation risk (Fig.3).

Spatial responses of intraguild prey in our study differed between the sexes, with female ferrets exhibiting greater avoidance of badgers than males. Female ferrets are potentially more vulnerable to predation than male ferrets because they often need to minimize the risk of predation on neonates; dens of maternal females frequently contain prey provisioned for offspring, and badgers are attracted to burrows containing dead prairie dogs (Biggins 2000). Similar to badgers, ferrets exhibit intersexual differences in social behavior and spacing patterns; female ferrets maintain smaller territories than males (Forrest et al. 1985; Jachowski et al. 2010; Livieri and Anderson 2012), presumably because females are focused on food resources and males are controlling access to potential mates (Powell 1979; Miller et al. 1996). The smaller territory sizes of female ferrets likely result in less frequent overlap and decreased potential for encounters with badgers relative to that experienced by male ferrets. A lower probability of engaging with intraguild predators likely decreases the probability of predation (Moehrenschlager et al. 2007). Sex-biased avoidance of predators by females with young is common in mammals [e.g., moose (*Alces alces*, Oehlers et al. 2011); bighorn sheep (*Ovis canadensis*, Berger 1991); cheetahs (*Acinonyx jubatas*, Durant 2000)], and indeed, antipredator behaviors have been documented to shape maternal behavior in other intraguild predator–prey systems (Waltzer and Schausberger 2011; Wen-San and Pike 2012). When establishing territories, female ferrets likely select for areas that minimize the risk of predation to young and also for areas that provide adequate prey.

Availability of food resources also influenced spatial interactions between intraguild predators and prey in our study system. We expected that avoidance of badgers by ferrets would decrease as densities of prairie dogs declined because ferrets would accept higher risks of predation in exchange for access to adequate food resources (e.g., Lima and Dill 1990, Thompson and Gese 2007; Wilson et al. 2010); however, our results suggested an opposite trend. Both sexes of ferrets avoided badgers when prey densities were relatively low, and both sexes exhibited less avoidance at relatively high prey densities; however, female ferrets maintained greater avoidance of badgers over a wider range of prey densities when compared with males (Fig.2). Decreased competition might explain why ferrets exhibited less avoidance of badgers as densities of prairie dogs increased. Hyperabundant food resources can relax interference competition and facilitate coexistence between two competitors (Holt and Polis 1997). Perhaps badgers exhibit less aggressive behavior toward ferrets when prairie dogs are abundant, and ferrets might respond by decreasing spatial avoidance. Although this relationship has not been well documented among carnivores, the abundance of food was suspected to lower competition and contribute to the tolerance of red fox by coyotes in Yellowstone National Park (Gese et al. 1996). Similarly, the amount of food eaten by dominant carnivores greatly influenced the level of tolerance exhibited toward other carnivores in Africa (Kruuk 1972; Schaller 1972).

Greater density of prairie dogs not only increases the amount of available food for ferrets, but the concomitant increase in prairie dog burrows also would lower risk of predation by badgers because of increased availability of refuges. Prey typically responds to predator presence by increasing use of refuges (Sih et al. 1988). Our results support a conclusion by Biggins et al. (2006c) that the positive association between security and food resources likely reduces the need for ferrets to trade off productive but risky habitats for less productive, safer ones.

Contrary to our expectations, age did not strongly influence models of space use by intraguild prey. We expected adult ferrets to exhibit greater avoidance of badgers than juveniles because other research has suggested a social hierarchy in this species (Forrest et al. 1988; Biggins et al. 2006a; Livieri and Anderson 2012). Perhaps social hierarchies in ferrets exist to provide control of food resources (by females) and mating potential (by males) more than avoidance of predators; future research could investigate this hypothesis.

We also expected that ferrets occupying smaller colonies would exhibit less spatial avoidance of badgers than ferrets on larger colonies because increased availability of space often is associated with lower predation risk in traditional and intraguild predator–prey systems (Suhonen 1993; Sergio et al. 2003; Kotler et al. 2004; Morrison et al. 2010). Our results did not support this prediction; however, our research focused on two colonies that were large (250.3 and 284.6 ha) at Buffalo Gap and 6 colonies that varied in size (9.3 to 207.7 ha) at Lower Brule; therefore, the variable for prairie dog colony size was likely confounded with study site, which was in our top model, but had confidence intervals around the parameter estimate that encompassed 0. Many other aspects that could influence spatial behavior of ferrets and badgers differed between the study sites, including surrounding habitat, alternative prey, predator community, proximity to roads, and other human activity. Consequently, our data might not have permitted a rigorous test of the effect of colony size on spatial interactions between badgers and ferrets.

Our results rely on a couple of important assumptions. One assumption is that we accurately characterized areas used by badgers. The number of locations used to estimate UDs for some badgers is slightly lower than 30, the number typically suggested to estimate the home range of an organism (Seaman et al. 1999). The UDs for 5 of 15 badgers were estimated with 27–29 locations. However, the remaining 10 badger UDs were estimated with 32–84 locations (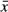 = 57). Second, we assumed that ferrets spatially avoided badgers rather than the opposite. Theoretically, the spatial patterns we described could be attributed to avoidance of ferrets by adult female badgers, perhaps because ferrets are superior at exploitative competition. Although this explanation is plausible, we believe that the more likely explanation for the space use patterns we documented is avoidance of badgers by ferrets to reduce risk of intraguild predation by badgers. Not only are badgers up to 8 times heavier than ferrets (Anderson et al. 1986; Minta 1992), but they are known to kill ferrets (Biggins et al. 1999, 2006a, 2011b). We estimated densities of prairie dogs on relatively small proportions of large prairie dog colonies as compared to small colonies, and increased sampling could have improved our estimates of relative densities of prairie dogs, especially on large colonies. Additionally, estimates of relative densities might have been improved by employing alternative methods such as spatially explicit capture–recapture models (Effords 2004), interpolation (e.g., Wilson et al. 2010), or counting burrows within grids (e.g., Eads et al. 2011). Our sample sizes of both ferrets and badgers were biased toward females – which might be expected in species that are intrasexually territorial – might have made it easier to detect trends for females because of increased statistical power. Larger and evenly distributed sample sizes would allow a more robust test of spatial patterns in males.

Our alternative hypothesis – that space use patterns of ferrets and badgers would overlap at fine spatial scales – was only supported when interactions between adult female badgers and ferrets occurred at relatively high density of prairie dogs. These results might appear to contradict recent research suggesting that badgers concentrate their activities in areas frequently used by ferrets and that they actively pursue ferrets (Eads et al. 2013). Several issues, however, hinder direct comparisons between these studies. First, our study used locations of individually marked animals, and consequently, we were able to determine differences in patterns of spatial interactions by sex and age categories. Second, radio transmitters on badgers in our study permitted collection of locations when individuals were active aboveground and resting belowground, while Eads et al. (2013) only used locations of badgers aboveground. Different space use patterns are likely to emerge when locations are obtained from animals engaged in differing activities and behaviors. Third, the studies differ in scale. Within a relatively short temporal scale, badgers might selectively excavate burrows in areas used by ferrets (Eads et al. 2013), but over longer time periods, our study revealed that some ferrets (females) are able to spatially avoid some badgers (adult females). We suggest that the conclusions of this study and Eads et al. (2013) are both valid, and that differences are due, in part, to activity of badgers when locations were collected, scale, and most importantly, the use of marked individuals. In our study, failing to account for sex would have obscured relationships that structure spatial interactions, and the asymmetric patterns of avoidance of badgers exhibited by ferrets would have been missed.

Our study has several implications for understanding spatial interactions between other sympatric species. First, we documented complex patterns of avoidance in an intraguild predator–prey system, but these patterns would likely have been undetectable if the sex of both predators had not been considered explicitly. Second, intersexual differences in patterns of space use also likely shaped spatial interactions between the species by creating differences in distribution of predation risk. Because reproductive strategies of males and females often result in sex-specific patterns of space use, this factor is likely to shape spatial interactions in many ecological systems. Third, density of food resources influenced observed spatial interactions between the two intraguild competitors, which suggests that the strength and direction of interactions will vary over space and time with extrinsic factors. These results support the need for interpreting spatial relationships in the context of both intrinsic and extrinsic influences on sympatric species.
